# Integrating molecular subtypes, genomics and functional dependencies to identify context-specific therapeutic vulnerabilities in small cell lung cancer

**DOI:** 10.1186/s40364-026-00991-3

**Published:** 2026-09-05

**Authors:** Ilaria Cassotti, Vito Fiume, Paolo Gandellini, Clelia Tiziana Storlazzi

**Affiliations:** 1 Department of Biosciences, University of Milan, Milan, Italy; 2 Department of Biosciences, Biotechnology and Environment, University of Bari Aldo Moro, Bari, Italy

**Keywords:** Small cell lung cancer, Cancer vulnerabilities, EcDNA, Mutation, Copy number variation, Synthetic lethality, Molecular subtype, Dependency screenings

## Abstract

Small cell lung cancer is one of the most aggressive malignancies, characterized by rapid tumor growth, early metastatic spread and extremely poor survival. Although most patients initially respond to platinum-based chemotherapy, relapse is almost inevitable and treatment options at recurrence remain limited. The recent introduction of immune checkpoint inhibitors has provided only modest clinical benefit, largely due to the fact that these tumors are immunologically cold. These limitations highlight the urgent need to better understand the molecular features of small cell lung cancer in order to identify more effective therapeutic strategies. In this review, we summarize current knowledge of the molecular landscape of small cell lung cancer, with particular emphasis on transcriptome-based classifications that have identified four major molecular subtypes defined by distinct transcriptional regulators and gene expression programs. We discuss how these classifications have improved the biological understanding of the disease and stimulated efforts to develop subtype-specific therapeutic strategies. At the same time, we highlight important limitations of this framework, including the remarkable transcriptional plasticity of tumor cells, which allows dynamic transitions between subtypes and may contribute to therapeutic resistance. To address these challenges, we examine additional molecular features that may represent more stable vulnerabilities, including recurrent genomic alterations, such as the widespread loss of tumor suppressor genes or oncogene amplifications through extrachromosomal DNA. We also discuss emerging approaches aimed at identifying novel context-specific cancer dependencies, including genome-scale functional screens in vitro and in vivo and genetic restraint analyses. Finally, we consider the growing potential of liquid biopsy strategies, which exploit the high level of circulating tumor DNA in patients with this disease to detect clinically relevant genomic alterations and monitor tumor evolution. Overall, this review highlights both the opportunities and challenges associated with molecular stratification in small cell lung cancer. The integration of transcriptional classifications with genomic and functional approaches may help identify more robust therapeutic vulnerabilities and guide the development of more effective treatments for this highly aggressive disease.

## Background

Small Cell Lung Cancer (SCLC) accounts for approximately 14% of all lung cancer cases [1]. It is the most aggressive form of lung cancer, characterized by rapid progression and high mortality. The five-year survival rate is approximately 7%, making it one of the deadliest malignancies. Median survival is typically around seven months. The primary risk factor for SCLC is tobacco use; indeed, over 95% of patients are either current or former smokers. Additional contributing factors include exposure to radon gas, air pollution and certain genetic predispositions [1, 2].

SCLC is marked by an aggressive progression, characterized by high genomic instability, rapid tumor proliferation, increased vascularization and a strong tendency for metastasis [2]. Consequently, at the time of diagnosis, the majority of patients exhibit metastatic spread beyond the thoracic cavity [3]. Despite decades of research, advancements in survival outcomes and therapeutic efficacy have remained limited, resulting in SCLC being classified as a “recalcitrant” cancer [4].

SCLC is primarily treated with systemic therapies, as surgical resection is generally reserved for highly localized disease. Chemotherapy remains the standard of care across all stages, with first-line treatments typically consisting of platinum-based cross-linking agents in combination with topoisomerase inhibitors administered over multiple cycles. In extensive-stage disease (ES-SCLC), radiotherapy is generally introduced in selected settings following initial chemotherapy [5].

Although SCLC typically shows an initial favorable response to treatment, a significant proportion of patients eventually develop resistance to chemotherapy [6]. Upon recurrence, the most commonly administered second-line therapy is topotecan, a topoisomerase I inhibitor [7].

Clinical progress over the past decade has been modest. The most significant advance has been the approval of immune checkpoint inhibitors (ICIs). However, only a small subset of patients (approximately 12%) achieve durable benefit and remain progression-free at one year following treatment [2]. This limited efficacy largely reflects the immunologically “cold” nature of SCLC, which is characterized by minimal immune cell infiltration. Moreover, reliable biomarkers capable of predicting response to immune checkpoint blockade have yet to be identified [8]. Beyond immunotherapy, no targeted therapies have been approved for SCLC and no disease-specific biomarker has been clinically validated to guide treatment selection. As a result, therapeutic decisions remain largely independent of the molecular features of individual tumors. A deeper understanding of the molecular characteristics of SCLC is thus essential for developing more effective and personalized therapeutic strategies.

In this review, we summarize the current transcriptome-based classification of SCLC subtypes, with particular emphasis on the challenges posed by transcriptional plasticity and the dynamic remodeling of gene expression programs. Given the limited efficacy of current treatments and the disappointing outcomes of ongoing clinical trials, we also examine the genomic landscape of SCLC to identify more stable molecular features that may support a more robust classification and uncover novel actionable vulnerabilities.

## Molecular subtypes of SCLC

**Fig. 1:**
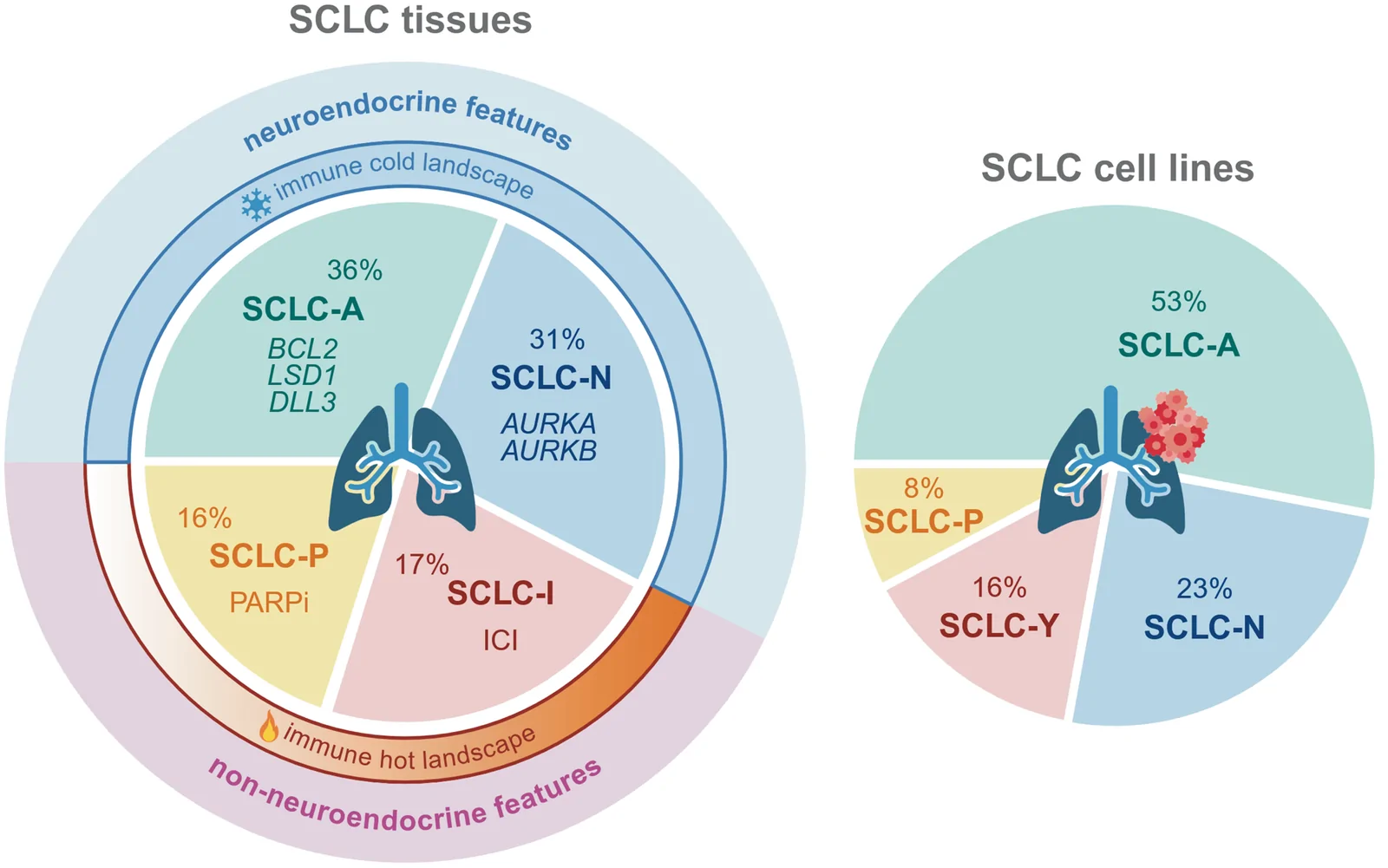
Comparative distribution of SCLC molecular subtypes across tissues and cell lines. Schematic overview of the four major small cell lung cancer (SCLC) subtypes (SCLC‑A, SCLC‑N, SCLC‑P, and SCLC‑Y/I) in patient tissues (*left*) and in 51 cancer cell line encyclopedia (CCLE) cell lines (*right:* SCLC-A *n* = 27, SCLC-N *n* = 12, SCLC-P *n* = 4, SCLC-Y *n* = 8). Pie charts show the relative frequency of each subtype based on Gay et al. (2021) [9] for tissue samples and on overexpression of the four lineage‑defining transcription factors for CCLE cell lines. In the tissue diagram, the outer rings depict the distribution of neuroendocrine versus non‑neuroendocrine features and the immune‑landscape gradient, highlighting higher immunogenicity in SCLC‑I than in SCLC‑P. Representative molecular vulnerabilities explored therapeutically for each subtype are indicated. Although several subtype‑specific targets have been identified, clinical responses remain variable, underscoring the complexity of translating SCLC molecular subtype biology into effective treatments

SCLC tumors have recently been classified into four molecular subtypes, each distinguished by the expression of a specific lineage-defining transcription factor (Fig. 1).

The neuroendocrine (NE) subtypes are characterized by expression of ASCL1 (SCLC-A) and NEUROD1 (SCLC-N) (Fig. 1), while POU2F3 (SCLC-P) has been recently recognized as a defining marker of a previously overlooked non-NE variant of SCLC [10]. A fourth proposed subtype, SCLC-Y, was originally associated with the expression of the transcription factor YAP1 [11]; however, subsequent studies have not consistently validated SCLC-Y as a distinct molecular category [12]. Notably, this fourth group lacks a clear transcriptional signature yet exhibits distinct gene-expression patterns, including elevated levels of immune checkpoints and human leukocyte antigens [9].

Importantly, emerging evidence suggests that the representation of SCLC subtypes may differ significantly between patient-derived tissue samples and established cell lines (Fig. 1). SCLC-A and SCLC-N are the most concordant across tissues and cell lines, with ASCL1 or NEUROD1 expression, respectively, prevalent in both tumor and cell line contexts, supporting their biological relevance and translational potential. SCLC-P classification based on POU2F3 expression is also consistent between tissue and cell samples; however, this subtype is underrepresented in cell lines [7, 13]. The difference between tissues and cell lines becomes more pronounced when examining the SCLC-Y subtype. YAP1 expression indeed shows inconsistent detection across tissue samples and cell lines (Fig. 1). Several studies report low prevalence and poor reproducibility of YAP1 in tumor biopsies [12]. This discrepancy has led to speculation that SCLC-Y may represent a cell line-specific phenotype or an artifact of in vitro culture conditions rather than a stable molecular subtype in vivo. Moreover, it was recently discovered that YAP1+ SCLC cell lines unexpectedly harbor *SMARCA4* mutations and exhibit transcriptional profiles more closely resembling those of thoracic *SMARCA4*-deficient malignancies than of SCLC [14], further calling into question the validity of the SCLC-Y subtype. This lack of consistency led to the classification of triple-negative SCLC tissues, defined by the absence of ASCL1, NEUROD1, and POU2F3, as the “inflamed” subtype, SCLC-I [9] (Fig. 1). Immunohistochemical profiling revealed that this subset is indeed characterized in vivo by a high density of CD8+ tumor-infiltrating lymphocytes. [15].

The four subtypes can be broadly grouped into NE and non-NE categories (Fig. 1). Both SCLC-A and SCLC-N subtypes exhibit elevated expression of NE signature genes, such as Chromogranin A (CHGA) and Synaptophysin (SYP). SCLC-A is typically associated with the classic morphological features of SCLC, consisting of cells that are typically small, with reduced cytoplasm, poorly defined borders and granular chromatin distributed throughout the nucleus. In contrast, SCLC-N typically exhibits a variant morphology, with larger cells characterized by more abundant cytoplasm, variable chromatin patterns and often prominent nucleoli. On the other hand, the RE1 Silencing Transcription Factor (REST), a known repressor of NE genes [16], is higher in SCLC-P and SCLC-I cells [9].

Tumors are not evenly distributed across subtypes, with the majority (36%) classified as SCLC-A, followed by SCLC-N (31%). In contrast, the SCLC-I and SCLC-P subtypes account for a smaller proportion of tumors, representing 17 and 16% of cases, respectively [9] (Fig. 1).

Recent findings have highlighted ATOH1, a neurogenic transcription factor, as overexpressed in a distinct subset of SCLC. Tumors expressing ATOH1 exhibit unique transcriptional signatures and show particularly aggressive traits, including enhanced metastatic potential [17]. Despite its biological relevance, there is ongoing debate as to whether ATOH1 defines a separate molecular subtype or not. While some studies support its classification as a novel subtype based on its distinct functional profile, others argue that its frequent co-expression with NEUROD1 together with the lack of an exclusive expression pattern suggest that it functions more as a modulator of the tumor phenotype rather than as a true classifier [18]. In this context, ATOH1 is better regarded as a biological program that refines the behavior of existing subtypes.

It has also been reported that, at the single-cell level, lineage-defining transcription factors in SCLC tumors are typically mutually exclusive, with most cells predominantly expressing a single transcription factor [12, 19].

Furthermore, recent insights into the molecular dynamics of SCLC suggest that tumor subtypes may represent dynamic transitions among cellular states [20]. It was further demonstrated that *MYC* overexpression can drive the temporal transition from SCLC-A to SCLC-N, ultimately culminating in the SCLC-Y subtype. This progression is driven by MYC-induced activation of the NOTCH pathway, reprogramming NE lineage commitment. Importantly, single-cell transcriptomics revealed that multiple subtypes often coexist within the same tumor, highlighting profound intra-tumoral heterogeneity [21]. These recent studies highlighting the plasticity of SCLC and the dynamic nature of its subtypes reveal new insights into therapeutic stratification and mechanisms of resistance.

## Genomic features of SCLC tumors

### Somatic mutations

SCLC is characterized by one of the highest tumor mutational burdens (TMB) among solid tumors, reflecting extensive genomic damage induced by tobacco carcinogens. In one of the largest cohorts analyzed to date, comprising approximately 3,600 SCLC cases, most tumors (99.5%) show microsatellite stability. TMB varied widely across cases, ranging from 0 to 276.3 mutations per Mb (mut/Mb), with a median of 7.8 mut/Mb and a mean of 9.5 mut/Mb; 38.9% of cases were classified as TMB-high (≥10 mut/Mb), and TMB distribution was similar across patients from diverse genetic ancestries [3]. In general, genomic alterations in SCLC can be broadly classified into point mutations, which primarily affect tumor suppressor genes (TSGs) and copy number alterations (CNAs), which involve amplifications of oncogenes or deletions of TSGs and may result in fusion genes [22]. All these genomic alterations are shared by both tumor samples and cell lines and function as driver oncogenic events [23, 24]. *ALK*, *ROS1*, and *RET* gene fusions are reported rarely, as they are mainly described as drivers in non-small cell lung cancer (NSCLC) [25–28]. In particular, *ROS1* and some *RET* fusions are observed after transformation from NSCLC to SCLC [27, 29]. Therefore, such alterations could not be considered as primary drivers of SCLC.

In the present review, driver genomic alterations will be reported in Tables 1 and 2, filtered by literature search using keywords such as “genomic alterations in SCLC,” “small cell lung cancer genomics”, “comprehensive genomic profiling of SCLC”, “molecular landscape of SCLC” and taking into account information included in the most comprehensive and influential genomic studies in SCLC reported to date [30, 42]. Table 1 reports gene mutation frequencies in SCLC patients, as documented in the literature, and derived from the 76 cell lines of the CCLE collection (accessed 7 July 2026). Loss‑of‑function events were identified using the “Damaging Mutations (Public 26Q1)” annotations, while gain‑of‑function events were assessed through the “Hotspot Mutations (Public 26Q1)” dataset. Among point mutations, nearly universal biallelic inactivation of *TP53* and *RB1* constitutes the defining genetic hallmark of SCLC [31]. These alterations are frequently accompanied by loss of heterozygosity at 17p and 13q, which harbor the *TP53* and *RB1* genes, respectively. Experiments in mice involving double knockout of *TP53* and *RB1* demonstrated the formation of tumors resembling human SCLC; these studies also showed that inactivation of *TP53* and *RB1* represents an early and necessary event in SCLC development [42].

**Table 1:** List of point mutations described in SCLC patients and cell lines and patients

Gene	Mutation Type	Functional Effect	Impact	Mutation Frequency (Patients)	Mutation Frequency (Cell Lines)	References
*TP53*	missense	loss/gain of function	BCL2 expression, apoptosis	92%	91%	[3, 30–32]
	deletion/truncation	loss of function	altered cell cycle progression, DNA damage response, apoptosis			
	splice-site	loss of function	altered cell cycle progression, DNA damage response, apoptosis			
	indel	loss of function	altered cell cycle progression, DNA damage response, apoptosis			
*RB1*	splice-site	loss of function	altered cell cycle progression, metastasis, apoptosis	73.5%	66%	[3, 14, 30, 33]
	missense					
	deletion					
	truncation					
*NOTCH1*	truncation	loss of function	neuroendocrine differentiation and cell proliferation	6%	3%	[3, 30, 34, 35]
	missense					
*NOTCH2*	missense	loss of function	neuroendocrine differentiation and cell proliferation	4%		[30, 34]
*NOTCH3*	missense	loss of function	neuroendocrine differentiation and cell proliferation	6%		[30, 34]
*KMT2D*	missense	loss of function	chromatin remodeling, transcriptional dysregulation	13%	17%	[3, 35]
	splice-site					
	indel					
	truncation					
*CREBBP*	deletion/truncation	loss of function	chromatin remodeling, transcriptional dysregulation	6%	5%	[3, 30, 36]
*PIK3CA*	missense	gain of function	cell survival, proliferation, and migration	6%	5%	[3, 30, 37]
*PTEN*	deletion	loss of function	cell survival, proliferation, and migration	10%	11%	[3, 30, 37, 38]
	truncation					
*KEAP1*	missense	loss of function	oxidative stress adaptation	3%	3%	[3]
*TET2*	missense	loss of function	transcriptional deregulation	2%	0%	[3]
	truncation					
*SMARCA4*	missense	loss of function	chromatin, remodeling, transcriptional dysregulation	1.5%	8%	[3]
	deletion/truncation

**Table 2:** List of recurrent CNA events described in SCLC patients and cell lines and patients

Cytoband	CNV Event	Key Gene(s)	Frequency (Patients)	Frequency (Cell Lines)	References
17pter-17p10	deletion	*TP53*	65%	40%	[3, 30]
13q10-13qter	deletion	*RB1*	40%	49%	[3, 30, 39]
3p14.3-3p14.2	deletion	*FHIT*	80%	62%	[3, 30]
3p12.3-3p12.2	deletion	*ROBO1*	80%	66%	[30]
10q10-10qter	deletion	*PTEN, DMBT1*	78%	47%	[40]
3q10-3qter	amplification	*SOX2*	55%	6%	[3, 39]
5pter-5p10	amplification	*CDH6, PC4, SKP2*	64%	13%	[3, 41]
1p34	amplification	*MYCL*	6–7%	32%	[3, 42, 43, 44, 45]
2p24	amplification	*MYCN*	2%	4%	[42–45]
8q24	amplification	*MYC*	6%	26%	[3, 42, 43, 44]

Another recurrent and functionally relevant mutational event involves the NOTCH pathway, a highly conserved cell-to-cell signaling system that regulates cell fate, differentiation and tissue homeostasis. Inactivating mutations in *NOTCH* genes are detected in approximately 25% of SCLC cases, most commonly affecting *NOTCH1* (3–7%), *NOTCH2* (3–4%), and *NOTCH3* (3–6%) [34]. These alterations suppress the NOTCH signaling pathway, which, in turn, promotes NE differentiation and cell proliferation [46]. Functionally, *NOTCH1* acts as a TSG in SCLC, restraining NE features and limiting tumor aggressiveness. Its loss promotes a highly NE phenotype and contributes to epithelial-to-mesenchymal transition and therapeutic resistance [46]. Epigenetic regulator genes are also frequently mutated, with loss-of-function alterations in *CREBBP* (5–6%) and *KMT2D* (13–17%) affecting histone acetylation and methylation, contributing to chromatin remodeling, transcriptional dysregulation, and lineage plasticity [36].

Additionally, mutations in genes involved in the PI3K/AKT/mTOR signaling cascade, a key regulator of cell growth and survival, occur in a smaller subset of SCLC cases but are increasingly under investigation for targeted therapy. Specifically, in a large cohort of 3,600 SCLC tumors, genomic profiling identified PI3K pathway alterations in a notable fraction of cases, including *PTEN* (9.9% of cases) and *PIK3CA* (5.6% of cases) [3]. These findings suggest that, although less frequent, activation of the PI3K/AKT/mTOR pathway represents a recurrent feature in a subset of tumors. Consistent with these data, an independent comprehensive genomic study of more than 50 SCLC cases identified alterations in this pathway. Interestingly, mutations in genes involved in the PI3K/AKT/mTOR pathway occur in a mutually exclusive manner [35]. Comprehensive analyses of 3,600 SCLC samples not only confirmed the near-universal biallelic inactivation of *TP53* and *RB1*, but also revealed a spectrum of less frequent, yet potentially functionally relevant, mutations. Among these, *KEAP1*, a negative regulator of the NRF2 antioxidant response pathway, was mutated in ~3% of cases, suggesting a role in oxidative stress adaptation; *TET2*, a DNA demethylase involved in epigenetic regulation, was altered in ~2% of patients, potentially contributing to transcriptional deregulation; *SMARCA4*, a SWI/SNF chromatin remodeler, was inactivated in ~1.5% of cases, often co-occurring with *CDKN2A* alterations and likely affecting chromatin accessibility [3]. Collectively, these findings expand the mutational landscape of SCLC, highlighting both the near‑universal loss of core TSGs and the presence of rare genetic alterations that may shape tumor heterogeneity, lineage plasticity, and therapeutic vulnerabilities.

### Copy number alterations and chromosomal instability in SCLC

Chromosomal instability is a hallmark of SCLC and underlies the marked heterogeneity observed among tumor cell populations. The SCLC genome is characterized by frequent replication stress and extensive DNA damage, leading to both focal aberrations and large-scale CNAs. Here, we describe the most frequent alterations in SCLC within both categories.

#### Large-scale genomic losses and gains

In Table 2 we summarize CNA frequencies for SCLC patient samples, as reported in the literature, and cell lines, as calculated from the CCLE Copy Number WGS Public 26Q1 (log2) dataset (accessed 7 July 2026). The frequency of each cytoband alteration was obtained based on the CNA of key target genes across the 53 annotated SCLC cell lines, considering that, in case of multiple gene candidates, we report the highest gene-specific frequency. Furthermore, gene-level copy-number status was defined using fixed thresholds, i.e. log_2_(CN_ratio + 1) values < 0.8 and >1.58 as deletions and amplifications, respectively.

The most recurrent chromosomal losses involve regions on 3p, 10q, 13q, and 17p, whereas recurrent gains occur on 3q, 5p, 6p, 8q, 17q, 19, and 20q (Table 2). These large-scale aberrations collectively shape the oncogenic landscape of SCLC: lost chromosomal regions harbor key TSGs such as *TP53*, *RB1*, and *FHIT*, while amplified regions harbor several oncogenes, including members of the *MYC* gene family [30]. Deletion of chromosome 3p is one of the most frequent and earliest genomic events in SCLC tumorigenesis. In a genomic study analyzing 110 SCLC samples, recurrent focal deletions at 3p14.3–3p14.2 were identified, encompassing the fragile-site tumor suppressor *FHIT*, and resulting in reduced expression and increased genomic instability. Additional focal losses were identified at 3p12.3–3p12.2, harboring the *ROBO1* gene [30]. Together, these early 3p alterations disrupt key tumor suppressor pathways and rank among the earliest structural changes driving SCLC development. Notably, studies in SCLC cell lines have shown that many of these interstitial 5q deletions arise through recombination between *Alu* repetitive elements. These *Alu*-mediated rearrangements contribute to large genomic deletions and underscore the profound genomic instability that characterizes SCLC [47]. Losses on the long arm of chromosome 10 are also recurrent genomic alterations in SCLC. Cytogenetic and microsatellite analyses showed loss of heterozygosity at 10q in up to 78% of primary SCLC samples [40].

Among the candidate genes on 10q, *PTEN* (10q23) has been identified as a critical TSG frequently inactivated in SCLC. Screenings of SCLC samples revealed *PTEN* deletions in approximately 11% of cell lines and 10% of primary tumors. Given that *PTEN* negatively regulates the PI3K/AKT signaling pathway, its inactivation is likely to promote uncontrolled cell proliferation and survival in SCLC [38]. Another candidate TSG on 10q is *DMBT1*, however, its role in SCLC pathogenesis remains to be fully elucidated [40]. Collectively, these recurrent chromosomal deletions suggest that additional TSGs, apart from those currently acknowledged, remain to be identified in SCLC. Although candidate genes have been proposed within these regions, their involvement remains largely speculative due to the lack of functional validation. Further characterization of these recurrent genomic alterations may therefore provide important insights into SCLC biology and reveal novel biomarkers and therapeutic targets.

Among recurrent chromosomal gains, 3q amplification leads to *SOX2* overexpression [39]. This genomic alteration is particularly enriched in the SCLC-A molecular subtype, where ASCL1 recruits SOX2 to transcriptionally activate NE effector genes such as *SMN1* and *WNT11*, thereby reinforcing NE identity [48]. Another frequent event is 5p gain (64%) [3], which includes the 5p13 amplicon harboring *CDH6*, *PC4*, and *SKP2*. In a panel of 22 SCLC cell lines, *SKP2* was amplified in 44% of samples, leading to p27^KIP1^ degradation and enhanced cell-cycle progression. Downregulation of *SKP2*, induced by antisense oligonucleotides, markedly suppressed SCLC cell growth, indicating that the gene plays an important role in SCLC proliferation [41].

#### Focal copy number increase through extrachromosomal DNA (ecDNA) amplification

**Fig. 2:**
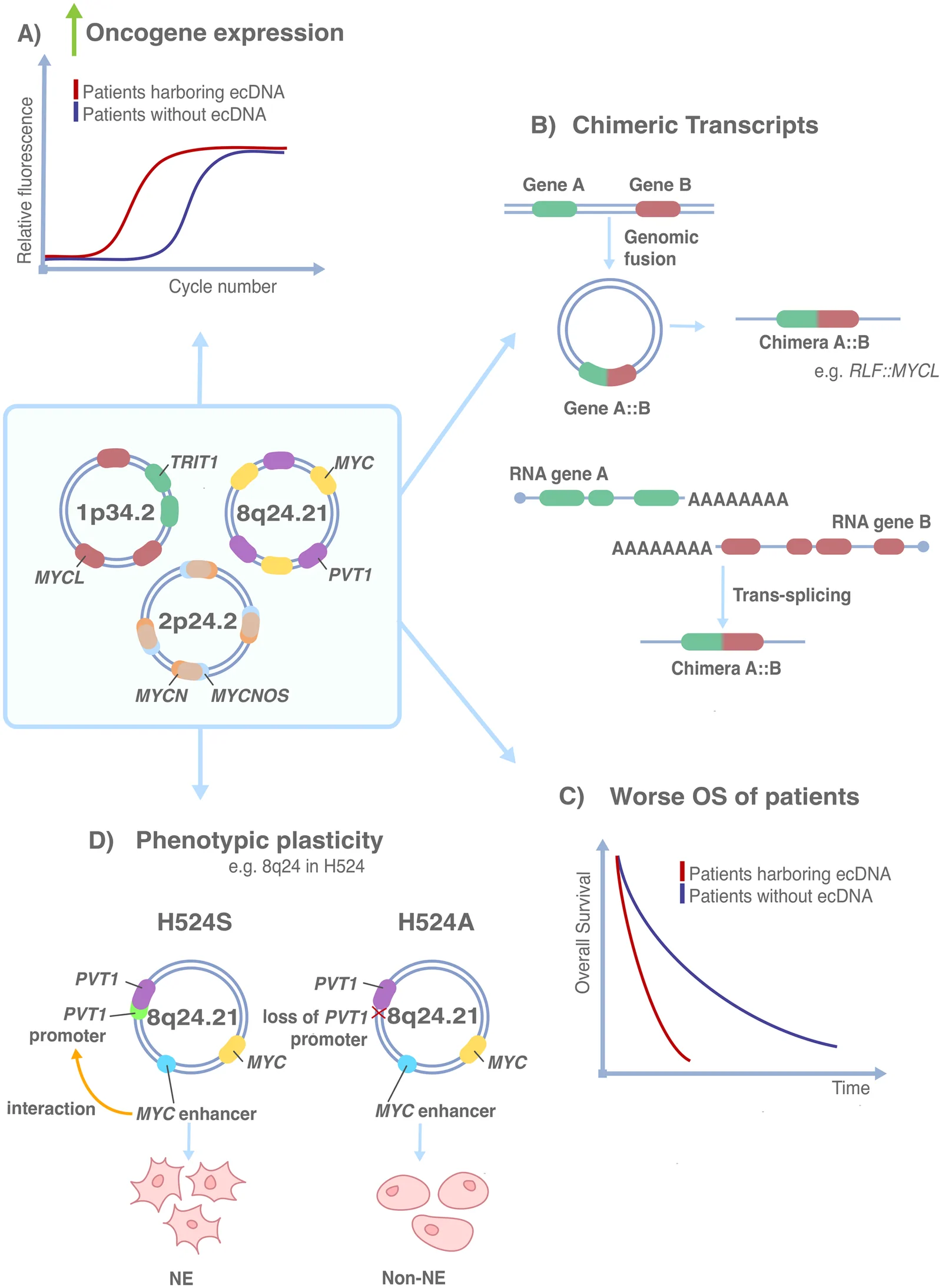
*MYC*-family gene ecDNAs in SCLC and their genomic, transcriptional and phenotypic consequences. *MYCL, MYC* and *MYCN* high-copy-number amplifications as ecDNAs are accompanied by the co-amplification of the close oncogenes *TRIT1, PVT1,* and *MYCNOS,* respectively, as shown in the central panel of the figure. ecDNAs can juxtapose distant genomic regions, thereby driving oncogene overexpression (**A**) and fusion genes and transcripts (**B**). In addition, these rearrangements may correlate with poorer overall survival (OS) in patients (**C**). Finally, enrichment of *MYC*-containing ecDNA has been reported to contribute to the NE phenotype (**D**) [43, 44]

In the context of genomic imbalance, amplifications of the *MYC* family oncogenes (*MYCL, MYCN*, and *MYC*) are a key feature of SCLC and play a central role in shaping transcriptional identity, proliferative behavior and therapeutic vulnerabilities [30]. High-level focal amplifications (>5 copies, occasionally > 50) of *MYCL*, *MYCN*, or *MYC* are observed in ~62% of cell lines (CCLE) and ~15% of primary tumors, confirming earlier estimates while revealing substantial heterogeneity in amplicon architecture [49]. Profiling of SCLC cell lines further showed that these high-level focal amplifications mainly occur as ecDNA, i.e., extra-chromosomal, head-to-tail, high-copy-number increases [50]. 28 of 32 amplified cell lines harbor ecDNA, and head-to-tail circular junctions were experimentally validated for both *MYCL* (NCI-H88 cells) and *MYC* (NCI-H524 [44], GLC1, GLC2, and GLC3 cells) (Fig. 2) [51].

*MYC*-family gene amplifications occur in a mutually exclusive manner, consistent with functional redundancy among *MYC* family members, except in the DMS-273 cell line, which harbors two distinct amplicons: one containing *MYC* and one containing *MYCL* [31, 49, 52, 53]. In a cohort of 58 SCLCs, focal amplifications involving *MYCL*, *MYCN*, or *MYC* were detected, with amplicon sizes ranging from 30 kb to 770 kb and frequently accompanied by co-amplification of adjacent genes (i.e., *MYCL* was co-amplified with *TRIT1* at 1p34.2 in 6 patients, *MYCN* with *MYCNOS* at 2p24.3 in 2 patients, and *MYC* with *PVT1* at 8q24.21 in 6 patients). For *MYCL* and *MYC*, these co‑amplifications have been attributed to complex intrachromosomal rearrangements that arise during amplification, resulting in the inclusion of neighboring genes on chromosomes 1 and 8, respectively [43].

ecDNA may arise through chromosomal breakage and circularization (episome model) [54–57], chromothripsis [58, 59], or breakage-fusion-bridge (BFB) cycles [60]. Their lack of centromeres leads to random mitotic segregation, aided by tethering to mitotic chromosomes through specific hypomethylated CpG-rich sequences known as ‘retention elements’ [61]. Such a phenomenon results in unequal oncogene copy numbers between daughter cells and fuels rapid clonal diversification [62].

#### Functional mechanisms and oncogenic fusion genes driven by ecDNA in SCLC

Recent large-scale whole-genome sequencing analyses have considerably expanded our understanding of *MYC* paralog amplification in SCLC, particularly highlighting the predominant role of ecDNA in driving these events [44, 50]. ecDNA represents a crucial genomic aberration resulting from CNA accompanied by complex intra- and inter-chromosomal structural variations, leading to oncogene deregulation in SCLC (Fig. 2).

Functionally, ecDNAs exhibit open, accessible chromatin, thus facilitating recruitment of the transcriptional machinery and resulting in high oncogene expression (Fig. 2A) [58, 63, 64]. Amplified oncogene upregulation could also result from ecDNA clustering within the nucleus to form hubs that promote interactions among multiple ecDNA molecules and with distal regulatory elements, or by hijacking distant enhancers, thereby rewiring the regulatory landscape in ways that would be impossible within the linear genome. ecDNA can also reintegrate into chromosomes, forming intra-chromosomal amplifications as homogeneously staining regions (HSRs), enabling dynamic shifts in oncogene dosage and regulatory configuration over time [65]. Altogether, these properties make ecDNA a powerful driver of rapid tumor evolution, phenotypic plasticity and therapeutic resistance. Given its biological relevance in cancer, ecDNA has been observed in approximately half of human tumors, with frequencies that vary widely across cancer types [66].

ecDNA enables novel juxtapositions of distally separated genomic loci in the linear genome, such as pairing *MYCL* with the regulatory gene *RLF* in the context of 1p34 amplification. These rearrangements create new enhancer–promoter interactions that markedly elevate oncogene transcription (Fig. 2A). For example, in NCI-H889 cells, the *RLF* enhancer displayed stronger activity than the *MYCL* promoter, whereas in NCI-H524 cells, *MYC* engages in potent enhancer–enhancer interactions with the adjacent *PVT1* locus. In addition to rewiring enhancer–oncogene interactions, ecDNA also serves as a major source of oncogenic gene fusions in SCLC (Fig. 2B). In particular, three ecDNA-positive SCLC cell lines (NCI-H889, NCI-H1092, and NCI-H1963) carry the *RLF::MYCL* fusion, generated by ecDNA-mediated rearrangements that reposition the *RLF* promoter upstream of *MYCL*, despite the two genes being ~259 kb apart and oriented on opposite strands in the linear genome [44]. The resulting 466 aa RLF/MYCL fusion protein is expressed at markedly higher levels than endogenous *MYCL* and enhances both tumorigenesis and metastatic dissemination [67]. Although *RLF::MYCL* is considered a recurrent fusion in 4.8% of 105 SCLC samples [67], a transcriptomic survey of 42 SCLCs (19 tumors and 23 cell lines) did not identify any fusion transcript present in more than two cases [43]. Instead, the study revealed recurrence at the level of 5′ fusion partners, most notably *RLF* and the long non-coding RNA *PVT1,* indicating preferential breakpoint usage rather than recurrent, tumor‑wide fusion events [43]. Table 3 summarizes the *RLF* and *PVT1* chimeras reported in SCLC cell lines, indicating their protein-coding potential and the amplification status of the corresponding 5′ and 3′ genomic regions encompassing each specific fusion partner.

**Table 3:** List of fusion transcripts in SCLC cell lines

5’ Fusion Partner	5’ Amplified Region	3’ Fusion Partner	3’ Cytoband	3’ Amplification	Cell Line	Protein Coding	References
*RLF*	1p34	*UBE2J2*	1p36	-	HCC-33	NO	[43]
*RLF*	1p34	*FAM132A*	1p36	-	HCC-33	YES	[43]
*RLF*	1p34	*MYCL*	1p34	+	NCI-H1963	YES	[43, 44]
*RLF*	1p34	*COL9A2*	1p34	+	NCI-H1963	NO	[43]
*RLF*	1p34	*BCL2L1*	20q11	+	NCI-H1963	NO	[43]
*RLF*	1p34	*HM13*	20q11	+	NCI-H1963	NO	[43]
*RLF*	1p34	*SMAP2*	1p34	+	NCI-H1963	YES	[43]
*PVT1*	8q24	*MYH7*	14q12	+	NCI-H82	Not determined	[43]
*PVT1*	8q24	*CHD7*	8q12	+	NCI-H2171	YES	[43, 68, 69]
*PVT1*	8q24	*SLC7A7*	14q11	-	NCI-H2171	Not determined	[43]
*PVT1*	8q24	*CCNB1IP1*	14q11	-	NCI-H2171	YES	[43]
*PVT1*	8q24	*CLVS1*	8q12	+	NCI-N417	Not determined	[43]
*PVT1*	8q24	*LY6H*	8q24	-	NCI-H446	Not determined	[43]
*PVT1*	8q24	*AKT3*	1q44	+	GLC1HSR	YES	[51]
*PVT1*	8q24	*EYA1*	8q13	+	GLC3	YES	[51]

Consistent with its frequent involvement as a 5′ breakpoint, *RLF* appeared as the upstream partner in seven distinct fusion transcripts detected across two SCLC cell lines (NCI-HCC33 and NCI-H1963). These fusions were highly heterogeneous (e.g., *RLF::UBE2J2*, *RLF::FAM132A*, *RLF::COL9A2*, *RLF::BCL2L1*, *RLF::HM13*, *RLF::SMAP2*, and *RLF::MYCL*), and only a subset were predicted to be protein‑coding. Importantly, some of the fusions were associated with co-amplification and genomic juxtaposition of the 5’ and 3’ partner genes. *RLF:: MYCL* is reported as recurrent in cell lines and tumor samples [39]. Similarly, *PVT1* appeared as the 5′ partner in six fusion events across four SCLC cell lines (*PVT1::MYH7* in NCI-H82 cell line, *PVT1::CHD7*, *PVT1::SLC7A7*, *PVT1::CCNB1IP1* in NCI-H2171 cell line, *PVT1::CLVS1* in NCI-N417 cell line, and *PVT1::LY6H* in NCI-H446 cell line) (Table 3) [43, 68, 69]. In all cases, the 5′ portion of *PVT1* was amplified, as well as three of the seven *PVT1* fusion partners (*MYH7*, *CHD7,* and *CLVS1*) [43]. Notably, ecDNA amplifications can generate fusion transcripts not only by transcribing fusion genes arising from genomic rearrangements, but also indirectly through post-transcriptional mechanisms such as trans-splicing [70] or cis-splicing between adjacent genes (Fig. 2B) [71]. In this context, *PVT1* produces a broad spectrum of chimeric RNAs, which have been described not only in SCLC [43, 70] but also in multiple myeloma [72], medulloblastoma [73], gastric cancer [74] and acute myeloid leukemia [57]. *PVT1* most commonly served as the 5′ fusion partner, often involving exon 1, suggesting either a breakpoint hotspot within intron 1 or a highly active donor splice site that promotes the formation of post-transcriptional chimeras [75]. The functional relevance of most *PVT1* chimeras remains largely unclear. Notably, *PVT1* exon 1 in the *PVT1::MYC* chimera has been reported to stabilize the partner transcript via an SRSF1-dependent mechanism, thereby increasing cancer cell survival [76]. In SCLC, the *PVT1::AKT3* fusion has been implicated in promoting tumor progression by enhancing oncogenic signaling, thereby increasing cellular proliferation and reducing apoptosis [77]. Overall, these data suggest that *PVT1* chimeras could be potentially biologically significant in SCLC, warranting further investigation into their mechanisms of origin and oncogenic roles.

#### The role of ecDNA in SCLC heterogeneity, drug resistance and cellular plasticity

As previously discussed, high-copy-number amplification of *MYC* family genes as ecDNA affects patient prognosis and therapy resistance.

In particular, Pongor et al. (2023) [44] highlighted the importance of defining the ecDNA amplicon architecture, defining complex ecDNAs as those harboring multiple focal amplifications connected by diverse SVs. They differ from simple ecDNA and non-ecDNA amplifications, as the latter show a single focal amplification connected either head-to-tail (simple ecDNAs) or head-to-head/tail-to-tail due to BFB cycles. Importantly, patients whose tumors contained complex ecDNAs showed significantly worse overall survival (OS) than those with non-ecDNA amplicons, which occur as intrachromosomal amplifications such as HSRs, indicating that ecDNA topology rather than gene copy number alone is a clinically relevant determinant of aggressiveness. (Fig. 2C) [44]. Furthermore, from a functional perspective, ecDNAs may be associated with phenotypic plasticity in SCLC. In NCI-H524 cells, which harbor 8q24 amplification on ecDNAs, suspension NE-like cells retain *MYC* ecDNA copies, including *PVT1* (Fig. 2D). In contrast, adherent non-NE cells lose *PVT1* promoter-containing ecDNA fragments, reduce MYC dosage and upregulate YAP1, suggesting an ecDNA-dependent switch between NE and non-NE states [44] (Fig. 2D). A representative autopsy case further underscored the profound spatial heterogeneity of ecDNA-driven amplification: different metastatic sites exhibited distinct *MYC* architectures, with lung and adrenal lesions carrying *MYC* on ecDNA at variable copy numbers, whereas cervical lymph node and liver metastases showed *MYC* integrated as HSRs. H3K27ac HiChIP Enhancer connectome analysis revealed that ecDNA-positive cells displayed stronger *MYC–PVT1* promoter–enhancer interactions, accompanied by higher *MYC* expression levels. Although all derived cell lines retained NE features, the high *MYC-*ecDNA-positive lung tumor line upregulated NEUROD1, indicating a shift to a NEUROD1-high state. This case illustrates how *MYC*-ecDNA can fuel intratumoral heterogeneity, enhancer rewiring, and NE cell-state transitions, thereby linking ecDNA structure to functional divergence in SCLC [44].

As mentioned above, ecDNA contributes to tumor heterogeneity and drug resistance [75]. This genomic abnormality is particularly frequent in relapsed patients and drug-resistant SCLC tumor samples. In particular, Choudhuri et al. (2024) [78] demonstrated that *MYC*-family gene ecDNAs were enriched in cross-resistant patient-derived xenografts (PDX) models derived from relapsed patients. However, the relationship between *MYC*-family gene amplification and tumor evolution during therapy was recently called into question by George et al. (2024) [79]. Based on the analysis of 160 tumors from 65 patients, they reported that *MYC*-family amplifications are frequently absent in the tumor founder clone and showed no evidence of selection under therapeutic pressure or in association with drug resistance [79]. Taken together, these data highlight ecDNAs as key drivers of increased oncogene dosage, chromatin accessibility, enhancer hijacking and transcriptional reprogramming in SCLC, thereby shaping intratumoral heterogeneity, fusion-gene formation, lineage plasticity, therapeutic resistance, and metastatic progression. Given the current discrepancies in observations regarding *MYC*‑family ecDNA and its influence on therapy response, a systematic investigation of both the structural and functional properties of ecDNA in SCLC is urgently needed. Such analyses, including potential correlations with treatment resistance, will be essential for clarifying disease biology and guiding the development of targeted therapeutic strategies.

Despite their potential clinical role in SCLC, ecDNA detection methods represent a pitfall in SCLC patient monitoring. In fact, ecDNAs are experimentally detected in tumor samples by metaphase Fluorescence in situ Hybridization (FISH) using gene-specific DNA probes, or by computational methods using short- and long-read whole genome sequencing, as well as the Assay for Transposase-Accessible Chromatin using sequencing (ATAC-seq), the latter exploiting the enhanced chromatin accessibility of ecDNAs [80]. However, these approaches require specialized computational pipelines and dedicated analytical tools, which currently limit their scalability and widespread implementation in routine diagnostic and prognostic workflows.

## Subtype‑Specific molecular vulnerabilities: clinical trials and limitations

Molecular subtype-based classification has provided an important framework for understanding SCLC biology and for identifying putative therapeutic vulnerabilities (Fig. 1). However, its clinical impact has been limited largely because transcription-based subtypes are highly plastic and can shift under therapeutic pressure [81, 82], thereby reducing their utility for durable patient stratification. As a result, several targets initially linked to specific subtypes, such as BCL2, LSD1, and DLL3, have entered clinical testing, but with variable success. In this section, we discuss these subtype-associated targets as therapeutic vulnerabilities, highlighting the clinical trial evidence supporting or challenging their actionability, as well as the limitations that underscore the need for more robust patient stratification strategies in SCLC.

Within the ASCL1-driven subtype, high *ASCL1* expression is frequently accompanied by *BCL2* overexpression, which contributes to apoptosis resistance [9]. Notably, in vitro treatment of SCLC-A and ASCL1+ NSCLC cells, as well as in vivo treatment of xenografts with BCL2 inhibitors revealed marked specificity, with ASCL1+ cancers responding far more strongly than their ASCL1- counterparts [83]. These findings suggest that BCL2 represents a vulnerability in ASCL1+ NE lung cancers and likely contributes to lineage-specific tumor survival (Fig. 1). However, in a phase I trial for the BCL2 inhibitor navitoclax in relapsed/refractory SCLC (*n* = 39), objective responses were rare and progression-free survival was short, underscoring the limited therapeutic benefit of BCL2 inhibition in this setting [84]. Importantly, the study enrolled unselected patients and *BCL2* expression was not used to guide treatment or interpret outcomes. As a result, there is no evidence supporting BCL2 expression as a predictive biomarker of response, despite its clear biological relevance in ASCL1‑driven SCLC.

Targeting the Lysine-specific histone demethylase 1A (*LSD1*) has emerged as another promising strategy for suppressing tumorigenesis in SCLC-A [85] (Fig. 1). ASCL1 plays a central role in maintaining NE identity and promoting tumor growth, as demonstrated by studies showing that its experimental knockdown significantly impairs SCLC cell proliferation [86]. Although the ASCL1 protein is not directly druggable, LSD1 inhibition offers an indirect route to suppress its expression. Notably, LSD1 blockade has been shown to activate NOTCH signaling, which in turn downregulates ASCL1 and inhibits tumorigenesis in vivo [85]. Irreversible LSD1 inhibitors, such as T-3775440 and Iadademstat (ORY-1001), have shown strong antitumor effects both in vitro and in vivo. These compounds disrupt LSD1 interaction with key transcriptional regulators, such as INSM1 and GFI1B, thereby reducing ASCL1 expression and impairing NE differentiation [86, 87]. Indeed, the phase II CLEPSIDRA trial, which investigated ORY-1001 in combination with platinum plus etoposide in patients with relapsed ES-SCLC, reported preliminary signs of clinical activity, with partial responses in 40% of evaluable patients and prolonged stable disease (>4 months) in an additional 20% [88, 89]. However, the study faced significant limitations, including severe hematologic toxicity from the drug combination, a small patient cohort (only 14 enrolled), and recruitment challenges exacerbated by the COVID-19 pandemic. Moreover, interpretation of the results is further complicated by the lack of patient stratification based on LSD1 expression, which makes it impossible to establish LSD1 as a possible predictive biomarker.

Another feature of the SCLC-A subtype is the overexpression of *DLL3*, encoding for a protein ligand that acts as an inhibitor of the Notch signaling pathway and which is directly regulated by ASCL1 [90, 91] (Fig. 1). A positive correlation between ASCL1 and DLL3 expression has been observed in surgically resected, early-stage SCLC specimens [92], further supporting the biological link between these markers. In this study, DLL3 expression was quantified by immunohistochemistry, with “high” expression defined as values above the cohort median. Given its consistently and robustly elevated expression in SCLC, DLL3 emerged as a promising therapeutic target [93]. In fact, in an early Phase I study, the DLL3-targeting antibody–drug conjugate rovalpituzumab tesirine (Rova-T) showed encouraging initial activity in patients with DLL3-high SCLC, with higher response rates reported in this population [91]. However, subsequent trials failed to confirm a durable clinical benefit. In the Phase III TAHOE study, which compared Rova-T with topotecan as second-line therapy in DLL3-high advanced SCLC, the trial was terminated early, after the interim analyses demonstrated shorter OS and greater toxicity in the Rova-T arm [93].

The bispecific T-cell engager tarlatamab received FDA approval in May 2024 for ES-SCLC progressing after platinum-based chemotherapy. In the phase I study, tarlatamab demonstrated a manageable safety profile (i.e., most adverse events were low-grade cytokine-release syndrome) and showed durable antitumor activity, with an objective response rate of 23%, a median duration of response of 12.3 months, and responses ongoing at one year in a subset of patients [94]. In the Phase 2 DeLLphi-301 trial, tarlatamab demonstrated clinically meaningful activity in previously treated SCLC, with an objective response rate of 40%, a median response duration of 9.7 months, and a median OS of 14.3 months, although cytokine-release syndrome remained the most frequent adverse event [95]. The subsequent Phase 3 DeLLphi-304 trial compared tarlatamab with standard chemotherapy (topotecan, lurbinectedin, or amrubicin) and showed a significant overall-survival benefit, lower rates of high-grade toxicity and improved patient-reported outcomes [96]. Tarlatamab has also been evaluated as first-line maintenance therapy in combination with PD-L1 inhibitors in the Phase 1b DeLLphi-303 study, where the regimen demonstrated manageable safety and preliminary antitumor activity consistent with its use in earlier-line settings [97]. Although DLL3 is highly expressed in SCLC and required for the activity of DLL3-directed therapies, current evidence does not fully support DLL3 expression level as a predictive biomarker of treatment response. An exploratory retrospective analysis of Rova‑T trials indicated an association between DLL3‑high status and higher response rates; however, subsequent studies failed to corroborate these findings. In contrast, although tarlatamab trials enrolled only DLL3‑positive patients, they did not identify a correlation between DLL3 expression intensity and treatment response [98].

SCLC-N often exhibits elevated expression of Aurora kinases A and B (AURKA/AURKB), key regulators of mitotic progression [99]. This molecular signature reflects increased mitotic activity and cell-cycle dysregulation, two typical features of the SCLC-N subtype, rendering it particularly vulnerable to Aurora kinase inhibition (Fig. 1). Supporting this vulnerability, preclinical and xenograft studies suggest that NEUROD1/*MYC*-high SCLC tumors exhibit selective sensitivity to AURKA/AURKB inhibitors, such as alisertib (AURKA inhibitor), barasertib, or AZD2811 (AURKB inhibitors), which impair tumor growth and induce polyploidy and apoptosis [100, 101]. However, intrinsic resistance to Aurora kinase inhibition frequently emerges, largely driven by elevated BCL2 expression. Indeed, while ASCL1-high SCLC-A models are strongly dependent on BCL2, NEUROD1-high SCLC-N tumors rely more heavily on AURKB activity; nonetheless, SCLC-N models that also express high BCL2 become refractory to AURKB inhibition, as BCL2 suppresses AZD2811-induced DNA damage and apoptosis. Notably, the combined inhibition of AURKB and BCL2 restores sensitivity in these preclinical models of resistance [102]. Moreover, a randomized Phase II trial evaluated alisertib in combination with paclitaxel as second-line therapy for refractory SCLC, showing a modest but statistically significant improvement in progression-free survival, although the combination was associated with increased toxicity. Later exploratory analyses examined MYC expression and alterations in cell-cycle regulators, including CDK6, RB1, RBL1, and RBL2, none of which had been used to select or stratify patients. These features were assessed using tumor RNA profiling and targeted sequencing of available samples. Tumors with high MYC expression or cell-cycle gene alterations appeared to benefit more from alisertib, suggesting a potential predictive value of these markers. However, since no thresholds were established and the findings were not validated, these genes cannot yet be used as treatment-guiding biomarkers [103].

The transcription factor POU2F3, which drives the SCLC-P subtype, has been implicated in enhancing responsiveness to PARP inhibitors (PARPi) [104] (Fig. 1). Moreover, overexpression of SLFN11, a DNA damage response protein that impairs homologous recombination and triggers replication stress, has emerged as a key determinant of PARPi sensitivity [105]. Its expression correlates with response to both chemotherapy and PARPi in SCLC cell lines and PDX [106]. A Phase I/II trial investigating the combination of the PARPi olaparib and temozolomide was conducted in patients with relapsed SCLC and demonstrated promising activity. PDX models from the same patients mirrored clinical responses and identified a molecular signature associated with treatment sensitivity, highlighting SLFN11 as a potential predictive biomarker, although clinical validation remains lacking [107].

Moreover, non‑NE SCLC cases, such as those driven by POU2F3 or YAP1, display activation of the Hippo‑YAP axis [108]. In line with this, SCLC‑I tumors exhibit enhanced Notch signaling together with increased YAP/TAZ activity [104, 109] (Fig. 1). While YAP1 expression is limited in SCLC patients, its knockdown in a cell line representative of the chemically induced SCLC-Y subtype was reported to reduce proliferation, a finding confirmed in xenograft models [110]. These molecular features may suggest increased susceptibility of these subtypes to YAP/TAZ-targeted therapies, although the therapeutic potential of direct YAP1 inhibition remains to be explored [111]. Small molecules that disrupt YAP-TEAD interactions, as well as FDA-approved drugs such as dasatinib, which indirectly inhibit the YAP/TAZ pathway by blocking Yes1 phosphorylation, have been reported to significantly inhibit cell proliferation and tumor growth in preclinical SCLC models [112].

Lastly, SCLC-I tumors, characterized by enriched immune cell infiltration, high MHC class I/II expression, IFN-γ signaling and STING pathway activation, appear to be the subtype most responsive to immune checkpoint blockade [113]. Multiple clinical trials are currently underway to further explore this therapeutic potential. Among landmark studies, the IMpower133 phase III trial evaluated the addition of atezolizumab (anti-PD-L1) to carboplatin and etoposide in treatment-naive patients with ES-SCLC, demonstrating a significant improvement in OS and leading to the first FDA approval of an immunotherapy for SCLC [113]. The MAURIS phase IIIb trial assessed the same regimen, with interim results confirming clinical activity and tolerability and survival and safety outcomes consistent with IMpower133 [114]. The CASPIAN phase III trial further expanded the immunotherapy landscape by investigating durvalumab (anti-PD-L1), tremelimumab (anti-CTLA-4), or both in combination with etoposide-platinum chemotherapy [115]. Patients were enrolled regardless of molecular subtype. Independent transcriptomic analyses (RNA sequencing) of publicly available SCLC cohorts have shown that tumors with higher expression of immune‑related genes, including *CD8A*, *CD4*, MHC class I/II and components of the antigen‑presentation machinery derive the greatest survival benefit from durvalumab‑based therapy, underscoring the importance of the tumor microenvironment in shaping immunotherapy efficacy.

Beyond PD-L1 blockade, emerging combination strategies are being explored to potentiate antitumor immunity. The association of atezolizumab with lurbinectedin, an oncogenic transcription inhibitor [116], has demonstrated enhanced first-line activity compared with atezolizumab alone, albeit with increased toxicity, suggesting that this regimen may represent a promising option for first-line maintenance therapy [117]. Preclinical studies further support the synergy between DNA-damage response inhibition and immune checkpoint blockade: combining PARPi, such as olaparib or talazoparib, with anti-PD-L1 therapies, enhances PD-L1 expression, promotes cytotoxic T-cell infiltration and alleviates immune exhaustion in resistant models [104, 118]. The SCLC-I subtype may be particularly receptive to such combinations. Clinically, however, the durvalumab-olaparib regimen tested in relapsed SCLC yielded limited survival benefit and modest efficacy, highlighting the need for more refined strategies to inform therapeutic choices [119].

For completeness, all clinical trials referenced in this section are cataloged in the comprehensive review by Arya et al. (2026) [120], which provides an updated overview of the SCLC therapeutic landscape.

## Clinical relevance of the early and non-invasive detection of genetic alterations in SCLC

In light of the heterogeneous therapeutic responses observed in SCLC, the identification of predictive biomarkers and the ability to dynamically monitor treatment response have become critical, underscoring the potential value of non-invasive strategies. Among these, liquid biopsy has emerged as a reliable, accessible and minimally invasive approach, enabling longitudinal assessment of disease evolution and therapeutic response [121]. This is especially relevant in SCLC, where adequate tumor tissue is often limited. Moreover, SCLC is characterized by extensive shedding of circulating tumor DNA (ctDNA) compared with most other solid tumors, making it particularly suitable for plasma-based analyses [122]. In this context, serial plasma sampling allows dynamic tracking of cell-free DNA (cfDNA), providing both qualitative and quantitative insights into tumor burden, treatment response, and disease evolution over time [123]. Within this framework, the CATS/ML43257 study evaluated the clinical relevance of longitudinal cfDNA monitoring in 32 patients with ES-SCLC treated with first-line chemoimmunotherapy [124]. Baseline cfDNA concentration, variant allele fraction (VAF), and tumor fraction (TF) were analyzed as continuous variables by Next Generation Sequencing (NGS) and shallow Whole Genome Sequencing (shWGS) and correlated with clinical outcomes. Although no significant differences in baseline cfDNA metrics were observed across molecular subtypes defined in tumor tissue (likely due to the limited sample size), higher baseline cfDNA levels were significantly associated with shorter progression-free survival (PFS) and OS. During treatment, cfDNA levels showed a marked reduction, followed by a significant increase with disease progression and then frequently returned to baseline. Importantly, early dynamic increases in cfDNA, particularly between baseline and on-treatment timepoints, were associated with a higher risk of death, underscoring the potential clinical value of early cfDNA kinetics [124]. Targeted NGS identified tumor-related mutations in nearly all patients at baseline, with *TP53* and *RB1* being the most frequently altered genes, consistent with the established genomic landscape of SCLC. Baseline VAF emerged as a strong prognostic biomarker, with higher values significantly associated with increased risks of disease progression and death. Longitudinal VAF analysis revealed a rapid and substantial clearance of tumor-associated mutations during treatment in a relevant proportion of patients, followed by re-emergence at disease progression. Both baseline VAF and dynamic changes during therapy were significantly associated with PFS and OS [124]. Notably, *PTEN* mutations were detected in three patients, of whom one had a high VAF (20–50%). Interestingly, this patient developed brain metastases at disease progression, consistent with previous evidence implicating *PTEN* alterations in brain metastatic dissemination of SCLC [3]. In parallel, shallow whole-genome sequencing, a well-established approach for quantifying the proportion of tumor-derived cfDNA in plasma [125], enabled a quantitative assessment of the TF. TF was detectable in the majority of patients at both baseline and progression. Higher baseline TF was associated with an increased risk of disease progression.

In contrast, longitudinal TF analysis identified two distinct patterns: patients who achieved complete TF clearance during treatment had significantly longer PFS than those with incomplete clearance. Moreover, early increases in TF during treatment were associated with higher risks of both progression and death, further supporting TF as a robust and clinically informative biomarker in SCLC [124]. Collectively, cfDNA concentration, VAF and TF provided complementary and partially overlapping information on tumor burden and disease kinetics. Exploratory multivariable analyses identified baseline cfDNA, VAF, and the number of metastatic sites as the most relevant predictors of PFS and OS, highlighting the clinical value of integrating multiple liquid biopsy–derived metrics for patient stratification and outcome prediction.

Beyond mutation-based cfDNA metrics, a major methodological advance in SCLC liquid biopsy has been provided by Sivapalan et al., who showed that integrating genome-wide CNAs with mutation-based ctDNA profiling markedly improves liquid biopsy monitoring in SCLC [126]. In particular, by combining tumor-derived SNV/indel data with a plasma aneuploidy score into a composite metric termed “cell-free tumor load”, and by rigorously filtering out clonal hematopoiesis–related variants, the authors detected recurrent plasma CNAs involving chromosomes 1p, 3, 5p, 8q, 10q, and 17p that closely mirrored tumor tissue profiles. Notably, cell-free tumor load dynamics enabled the identification of distinct molecular response patterns strongly associated with OS, PFS and anticipated radiographic response. These metrics outperformed mutation-only approaches, although prospective validation is required given the retrospective design, small cohort size, and methodological heterogeneity [9, 126, 127].

Liquid biopsy approaches can also capture higher-order genomic features central to SCLC biology, such as ecDNA. Cell-free circular DNA can be detected in plasma and exhibits greater stability than linear cfDNA, potentially owing to its circular structure and reduced susceptibility to exonuclease degradation [128]. These properties support its potential as a liquid biopsy analyte, although robust clinical validation remains limited. In SCLC, current evidence largely relies on indirect inference of ecDNA from focal oncogene amplifications detected in cfDNA and comparison with matched tumor tissue, rather than direct isolation of circular DNA species. In a cohort of 82 patients with ES-SCLC, cell-free chromatin immunoprecipitation followed by sequencing identified *MYC* and *MYCL* ecDNA signals in 5 cases (6.1%), with amplification profiles closely matching those observed by whole-genome sequencing of tumors, supporting the feasibility of detecting ecDNA-derived signals in plasma [44]. However, current cfDNA workflows are not specifically optimized for ecDNA enrichment or structural characterization, and dedicated approaches, such as Circle-Seq or long-read sequencing, remain largely underdeveloped in SCLC.

Another important consideration is that ecDNA captures only the extrachromosomal component of the tumor genome and may not be detectable in all patients before treatment. Although preclinical studies have implicated *MYC*-family ecDNAs in systemic therapy resistance [78], their clinical significance remains to be established. Consequently, whether circulating ecDNA harboring *MYC*-family amplifications can predict prognosis, treatment resistance, or therapeutic response remains an important area for future investigation. If ecDNA-associated oncogene amplifications prove to represent actionable dependencies or reliable predictors of treatment response, ecDNA analysis could support patient stratification and guide targeted therapies. Moreover, the diversity in ecDNA number, size, structure and genomic content suggests that, when integrated with complementary cfDNA features such as mutation, methylation, or fragmentation profiles, circulating ecDNA may further improve the molecular subtyping of SCLC [129]. Overall, while circulating ecDNA shows considerable promise as a prognostic and/or predictive biomarker, substantial work remains to establish its clinical utility in ctDNA-based liquid biopsy strategies for SCLC, particularly for disease stratification, treatment monitoring and prediction of therapeutic resistance.

**Fig. 3:**
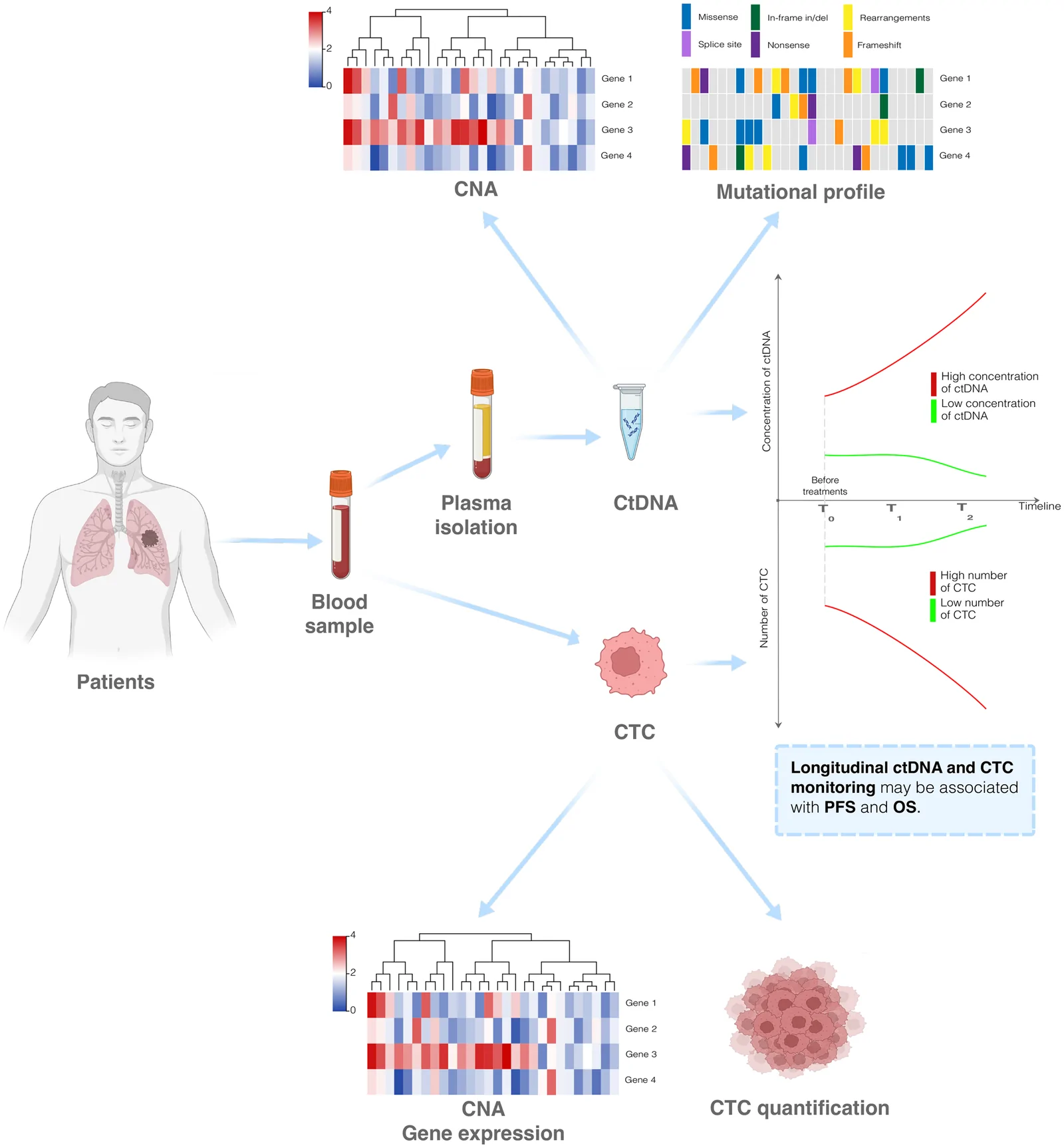
Schematic liquid biopsy approaches in SCLC integrating parallel analysis of ctDNA and CTCs. ctDNA is assessed for mutational profile and CNA, while CTCs are evaluated for enumeration, CNA, and expression profile. Longitudinal monitoring of both biomarkers correlates with clinical outcomes, with higher ctDNA levels and CTC counts (at T₀ and during treatment) being associated with shorter PFS and OS [124, 129]

While ctDNA-based approaches provide dynamic, highly sensitive measures of tumor burden and treatment response, complementary information on tumor biology and dissemination can be obtained from analysis of circulating tumor cells (CTCs), another key component of liquid biopsy strategies in SCLC (Fig. 3). Notably, CTCs are detected at remarkably high frequency in patients with SCLC, reflecting the aggressive biology and high metastatic potential of this disease. Since the first report by Kularatne et al. in 2002 [130], multiple studies using both epitope-dependent and epitope-independent enrichment strategies have consistently shown that 70–95% of patients with SCLC harbor detectable CTCs, a proportion substantially higher than that observed in most other solid tumors [130, 131]. This abundance makes SCLC a particularly suitable model for investigating the clinical and biological significance of CTCs. Despite their high prevalence, the role of CTCs as screening or early diagnostic biomarkers in SCLC remains largely unexplored. Low-dose CT screening has demonstrated limited efficacy in detecting early-stage SCLC, with most cases diagnosed at an extensive stage [132]. While preliminary evidence in NSCLC suggests that CTC detection may precede radiological tumor development in high-risk individuals, analogous data are currently lacking for SCLC [133].

In contrast, substantial evidence supports the prognostic value of CTC enumeration in SCLC. Multiple independent studies have shown that higher baseline CTC counts are strongly associated with shorter PFS and OS, irrespective of disease stage or metastatic burden [131, 134]. Although proposed cut-off values vary widely, ranging from 2 to over 100 CTCs per 7.5 mL of blood, baseline CTC count consistently emerges as an independent prognostic biomarker, with elevated levels being associated with markedly increased risks of disease progression and death [131, 135]. However, the absence of a standardized, universally accepted threshold remains a major barrier to clinical implementation [136]. Beyond enumeration, the biological organization of CTCs provides additional prognostic insights. Circulating tumor microemboli, defined as clusters of CTCs often associated with platelets or stromal cells, are detected in a subset of patients and correlate with particularly poor outcomes. These clusters are thought to promote immune evasion and confer protection from cytotoxic agents, thereby linking CTC aggregation to therapeutic resistance [137]. Importantly, baseline CTC count does not appear to predict treatment response [138]. Instead, dynamic changes in CTC levels during therapy provide more clinically informative insights. Several studies have demonstrated that a rapid and pronounced reduction in CTC count after the first cycle of chemotherapy is associated with improved survival, whereas persistently elevated or increasing CTC numbers identify patients with unfavorable outcomes [139–141]. In this setting, longitudinal CTC monitoring may serve as an early pharmacodynamic biomarker, enabling the identification of non-responders before radiographic progression. Molecular characterization of CTCs has further expanded their clinical relevance. Single-cell genomic profiling has revealed that baseline CNA patterns can accurately distinguish chemosensitive from chemorefractory disease. Notably, CNA-based classifiers predict intrinsic chemotherapy resistance but do not shift at relapse in initially chemosensitive patients, suggesting that the genetic determinants of primary resistance differ from those driving acquired resistance. This observation supports a model in which non-genetic mechanisms, such as transcriptional plasticity or epigenetic reprogramming, contribute to disease relapse [142].

Beyond genomic profiling, the evaluation of CTC expression patterns is emerging as a complementary strategy for investigating tumor heterogeneity and identifying clinically relevant cellular subpopulations [143]. However, despite extensive investigation, no studies have yet reported comprehensive transcriptomic profiling of CTCs in SCLC. Current evidence is limited to targeted analyses of selected genes and proteins. In particular, recent studies have shown that CXCR4 and JUNB expression in CTCs from treatment-naïve patients is associated with reduced OS [144]. CXCR4 is a G protein-coupled receptor involved in tumor growth, metastasis, and cell survival [145], while JUNB is an AP-1 family transcription factor that regulates genes implicated in invasion and metastasis and has also been reported to promote PD-L1 expression [146]. Notably, these analyses were performed following EpCAM-independent enrichment via Ficoll density-gradient centrifugation, enabling the recovery of heterogeneous CTC populations, including cells undergoing epithelial-to-mesenchymal transition, which may be underrepresented by EpCAM-based capture methods [144]. These findings suggest that transcriptional and protein profiling of CTCs may provide additional prognostic information beyond CTC enumeration and genomic alterations. However, further validation in larger prospective cohorts and standardized analytical workflows is required before clinical implementation.

Overall, cfDNA and CTC-based liquid biopsy approaches provide complementary and biologically informative views of tumor burden, evolution and therapeutic response in SCLC. Their high sensitivity, dynamic nature and strong prognostic value support their use as stratification and monitoring tools in clinical trials and translational research. Despite the strong biological rationale and growing clinical evidence, translating these approaches into routine clinical practice remains constrained by a lack of methodological standardization. Harmonization of pre-analytical workflows, bioinformatic pipelines, and, critically, the definition of validated thresholds for ctDNA dynamics, plasma aneuploidy scores and CTC enumeration will be essential to ensure reproducibility, cross-study comparability and robust clinical implementation. Addressing these challenges through standardized studies will be a key step toward fully integrating liquid biopsy into precision oncology strategies for SCLC (Fig. 3).

## New avenues for context-specific vulnerabilities

The challenges of treating SCLC reflect its biological complexity and underscore the urgent need for more effective targeted therapies. Although molecular subtypes have provided a framework for investigating genetic and therapeutic vulnerabilities, emerging evidence of SCLC plasticity and intra-tumoral heterogeneity calls into question the stability of an already debated classification. These dynamic features have important implications for therapeutic stratification and mechanisms of resistance. Consequently, relying solely on subtype identity to guide personalized treatment may be insufficient (Fig. 1), particularly because proposed therapeutic targets have not yet demonstrated robust predictive value and, in many cases, have not been systematically evaluated as true biomarkers of treatment response. Integrating genomic and transcriptomic profiling into this framework may therefore provide a more precise and actionable approach to identifying patient-specific vulnerabilities.

**Fig. 4:**
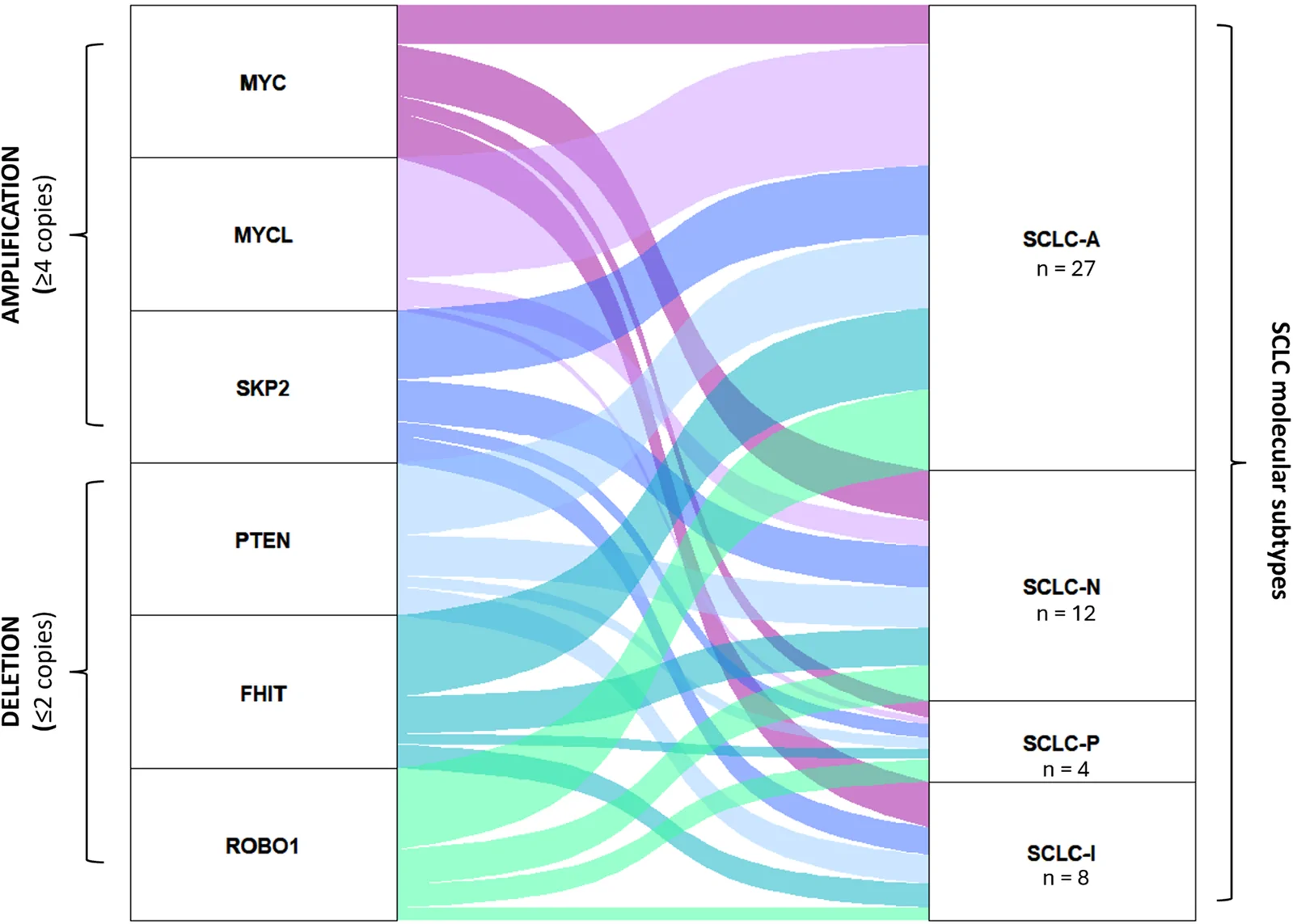
Genomic alterations in SCLC do not segregate by molecular subtype. Alluvial plot showing the frequency and distribution of major CNAs in CCLE SCLC cell lines grouped by molecular subtype (based on the classification reported in Ireland et al. 2020 [21]). Each color represents a specific gene, and the width of each flow reflects the frequency of that alteration within a given subtype. Data were obtained from the 51 SCLC cell lines in the CCLE (DepMap *copy number WGS Public 25Q3 (log₂-transformed)* dataset, accessed 6 October 2025). Subtypes include SCLC‑A (*n* = 27), SCLC‑N (*n* = 12), SCLC‑P (*n* = 4), and SCLC‑Y/I (*n* = 8), where *n* indicates the number of CCLE lines assigned to each subtype. Amplifications were defined as relative_copy_number ≥ 1.58 and deletions as relative_copy_number ≤ 0, based on the DepMap log_2_(CN_ratio + 1) metric. Statistical differences across subtypes were evaluated using a chi‑square test of independence. Significant differences were observed for *MYCL* (*p* = 1.7 × 10^− 8^), *PTEN* (*p* = 0.0022), *FHIT* (*p* = 6.2 × 10^− 5^), and *ROBO1* (*p* = 6.5 × 10^− 5^), all of which were enriched in the SCLC-A group, while *MYC* (*p* = 0.38), *MYCN* (*p* = 0.57) and *SKP2* (*p* = 0.095) did not show subtype‑specific enrichment. The substantial overlap in genomic alterations across subtypes highlights that, although certain trends reach statistical significance, transcription‑based classifications do not correspond to distinct DNA‑level alteration patterns

The rationale for identifying new vulnerabilities in SCLC should emphasize context specificity, focusing on tumor features that can stratify SCLC into biologically meaningful groups. A potentially robust approach might be to classify tumors based on stable genomic characteristics, such as recurrent mutations, amplifications, or deletions, which are less prone to change than transcriptional signatures and are often retained or even selected at relapse following therapy [79]. Notably, the association between molecular subtypes and genomic backgrounds is not straightforward (Fig. 4). Analysis of CCLE cell lines shows that most recurrent copy‑number events are broadly shared across SCLC‑A, SCLC‑N, SCLC‑P, and SCLC‑I, with only a minority displaying subtype‑restricted enrichment. Although certain *MYC* paralog amplifications show enrichment in specific transcriptional subtypes, such as *MYCL* in SCLC‑A and *MYC* in SCLC‑N/P (Fig. 4) [81, 147], these associations are not exclusive and the same alterations occur across multiple molecular subtypes. This lack of concordance indicates that transcription‑based subtypes and genomic backgrounds capture distinct and only partially overlapping dimensions of SCLC biology. Among potentially interesting genomic alterations, amplifications of *MYC* paralog genes define distinct subsets of SCLC. *MYC* amplification, in particular, has been shown to drive distinct biological and metabolic programs and to confer different therapeutic sensitivities [148].

Targeting MYC may therefore represent a promising therapeutic strategy in selected contexts. However, direct MYC inhibition has long been challenging due to its intrinsically disordered protein structure, which limits the formation of stable drug-binding sites [149]. Novel agents, such as OMO-103, a 91-amino acid miniprotein that disrupts MYC-MAX dimerization, may offer potential solutions. OMO-103 has entered early-phase clinical trials for various solid tumors, but it has not yet been tested specifically in SCLC [150]. Importantly, MAX interacts with all three MYC paralogs, but it shows lower binding affinity for MYCL than for MYC [151], suggesting that tumors driven by MYCL, which represent a sizable fraction of SCLC cases, may not benefit equally from OMO-103. Moreover, the functional consequences of *MYCL* and *MYCN* amplifications remain largely unknown, as no genetic or pharmacological vulnerabilities specifically associated with these alterations have yet been defined. Future investigation should also assess whether the molecular profile and vulnerabilities associated with *MYC* paralog amplification may vary depending on the rearrangement mechanism (i.e., focal amplification vs. ecDNA). In addition, *MYC/MYCL/MYCN*-ecDNAs warrant attention for their specific role in SCLC tumorigenesis or progression, particularly if their presence is mutually exclusive with other somatic mutations or CNAs, suggesting that they occur as a context-specific alteration.

From a therapeutic perspective, the direct targeting of driver genomic alterations is often not feasible, either because these genes are intrinsically undruggable or because their druggability has not yet been systematically assessed. MYC itself required decades of effort before a first-generation inhibitor entered clinical testing [150], and druggability predictions (e.g., CanSAR [152]) still classify MYCL and MYCN proteins as undruggable.

Alternatively, collateral dependencies arising from selected genomic alterations can be leveraged to identify vulnerabilities that are both biologically meaningful and clinically actionable. SKP2 exemplifies this principle. Although SKP2 is not a classical oncogenic driver, it represents a context‑specific vulnerability that emerges in *RB1*‑deficient tumors. SKPin‑C1, a selective small‑molecule inhibitor of the SCF (Skp2/Cks1) ubiquitin ligase complex, induces growth inhibition in both human and murine SCLC models lacking RB1. In contrast, *RB1*‑proficient NSCLC cell lines are at least one order of magnitude less sensitive. Genetic deletion of *SKP2* blocks tumorigenesis in *Rb1*/*Trp53*‑mutant lung models and is synthetically lethal with *RB1* loss, highlighting a dramatic dependency. In xenografts, systemic SCF (Skp2/Cks1) inhibitors reduce SCLC growth by ~55% and prolong survival, outperforming MLN4924 in some *RB1*‑null contexts, while the opposite pattern is observed in *RB1*‑wild‑type NSCLC [153, 154].

**Fig. 5:**
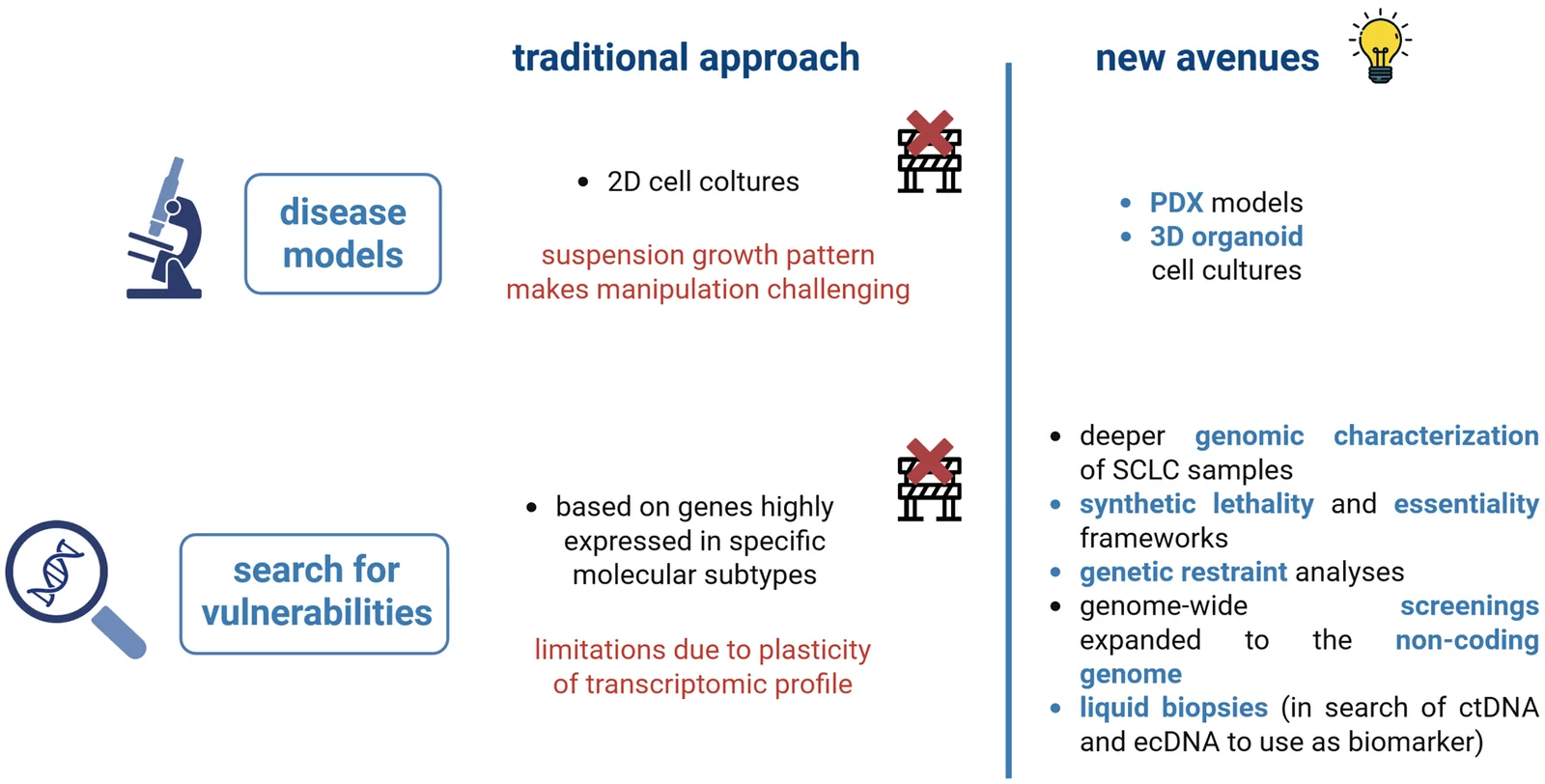
Pitfalls and emerging strategies in SCLC research. Schematic overview of the major challenges in SCLC research, highlighting the limitations of current cell models and the obstacles to identify novel therapeutic vulnerabilities. Emerging approaches aimed at overcoming these barriers and supporting the development of more tailored treatments for patients are highlighted

Context‑specific vulnerabilities can be uncovered through complementary genetic frameworks, including synthetic lethality and synthetic essentiality (Fig. 5). Synthetic lethality refers to gene pairs in which combined disruption is lethal, whereas alteration of either gene alone is tolerated. In contrast, synthetic essentiality refers to genes that become indispensable only in the presence of a specific oncogenic alteration. Considering both frameworks together broadens the landscape of potential therapeutic targets [155]. Synthetic lethality has already proven clinically valuable; for example, PARP inhibitors have shown efficacy in *BRCA1/2*-mutated cancers [156]. In SCLC, similar strategies could target recurrent genomic alterations, such as *TP53* or *RB1* loss or *MYC* paralog amplification, by identifying partner genes whose inhibition selectively kills tumor cells while sparing normal tissue. An illustrative example is the dependency of *MYC*‑amplified SCLC on AURKA, which stabilizes MYC protein and sustains its transcriptional activity. In MYC‑high tumors, AURKA inhibition preferentially impairs tumor cell viability while having limited effects on MYC‑low cells, representing a form of synthetic essentiality, a context-dependency in which an oncogenic alteration renders a normally non‑essential gene critical for tumor cell survival. Rather than arising from a strict digenic synthetic lethal interaction, this vulnerability emerges because the oncogene rewires cellular networks, creating a selective reliance on a cooperating factor that is usually not required in normal tissues [157]. It is also important to note that nearly all synthetic lethal phenotypes are not strictly digenic but rather polygenic and are often influenced by broader network effects and environmental factors [158]. This complexity must be taken into account when searching for vulnerabilities in SCLC.

Furthermore, synthetic lethal interactions frequently exhibit incomplete penetrance [159], meaning that not all patients harboring a given genetic alteration will respond effectively to inhibition of the corresponding partner gene or pathway. Such insights reinforce the idea that synthetic lethality in cancer is best understood as a network‑level phenomenon, which requires integrating genomic, transcriptomic and environmental factors to identify robust, clinically actionable dependencies.

A computational approach to identify dependencies and synthetic interactions is represented by genetic‑restraint analyses, which aim to detect genes under negative selection in specific biological contexts (Fig. 5). For example, genes that exhibit little to no deletion or deleterious mutation in cells characterized by specific genetic alterations (e.g., amplification of *MYC* paralogs or loss of *TP53/RB1*) are likely to be synthetic essentialities. Robust identification of these genomic patterns in SCLC requires large cohorts with deep and comprehensive sequencing. However, the number of fully profiled SCLC cases available to date remains limited. The largest dataset currently available, comprising ~3,600 tumors reported by Sivakumar et al. (2023) [3], relies exclusively on targeted NGS of 358 genes. Consequently, key genes that are uniquely relevant to SCLC biology may fall outside the scope of interrogation. Expanding genomic datasets will therefore be critical to uncover additional synthetic interactions and vulnerabilities. A recent pan‑cancer analysis illustrates the potential of this strategy by identifying recurrent patterns of co‑occurrence and mutual exclusivity among *MYC* paralogs across multiple tumor types [45]. In fact, several *MYC*‑associated genomic configurations emerged, including strong mutual exclusivity with specific oncogenic drivers such as *FGFR3* mutations in bladder cancer, *KRAS* mutations in lung adenocarcinoma, *PTEN* alterations and *FGFR2* mutations in uterine cancer. Within the lung neuroendocrine subset, *MYCL* amplification also appears mutually exclusive with *EGFR* mutations, suggesting distinct biological programs and potentially divergent therapeutic vulnerabilities in *MYCL*‑high versus *EGFR*‑driven tumors. These emerging patterns highlight how mutually exclusive alterations can reveal synthetic essentialities and context‑specific dependencies that may be therapeutically exploitable. Pursuing similar analyses in expanded SCLC cohorts will be essential for systematically mapping such vulnerabilities and guiding the development of targeted interventions.

Functional dependencies can be experimentally uncovered through genome‑wide screening strategies (Fig. 5). High‑throughput technologies such as CRISPR/Cas9 or RNAi enable simultaneous interrogation of thousands of genes, thereby revealing essential pathways and potential therapeutic targets at scale [160]. In SCLC, the application of these approaches has been limited, but recent studies demonstrate a growing interest in leveraging functional genomics to map subtype‑specific vulnerabilities. Duplaquet et al. (2024) [161] performed a genome‑wide CRISPR‑Cas9 screen in *POU2F3*‑positive SCLC cells, identifying the mSWI/SNF chromatin‑remodeling complex as a critical dependency and revealing *BRD9* and *SMARCA4/2* as targetable vulnerabilities. Similarly, Patel et al. (2026) [162] used a CRISPR/Cas9 screen to identify *HDAC3* as a radiosensitizing target in SCLC, demonstrating how loss of *HDAC3* enhances radiation response and highlighting epigenetic regulators as therapeutic candidates. Together, these examples illustrate how functional genomic screening has become a powerful strategy for mapping essential gene networks and revealing subtype‑specific vulnerabilities.

Importantly, these approaches should not be restricted to protein-coding genes. Increasing evidence from multiple tumor types demonstrates that long non-coding RNAs (lncRNAs) play critical roles in oncogenesis, therapy resistance and tumor progression [163–166]. Consistently, genome-scale functional screening approaches have been successfully applied to identify lncRNA dependencies across multiple cancer types [167, 168], facilitated by the development of RNA-editing enzymes, such as Cas13 and CasRx. For example, CRISPR-based loss-of-function screens have discovered essential lncRNAs involved in proliferation and survival in lung, breast, and hematological malignancies [169–171]. However, a systematic mapping of lncRNA dependencies of SCLC cells is still lacking.

Focusing on lncRNAs is of particular interest because they are amenable to therapeutic targeting. Antisense oligonucleotide-based strategies can selectively silence both nuclear and cytoplasmic lncRNAs via RNase H–mediated degradation, providing high target specificity and making them attractive candidates for therapeutic intervention [172, 173]. For example, we showed that gapmer antisense oligonucleotides designed to cleave *NFYC-AS1*, the antisense RNA transcribed head-to-head with the *NFYC* transcription factor at the 1p34 locus, significantly affect the viability of NCI-H82 SCLC cells [164]. Moreover, given that a vast proportion of the human transcriptome is non-coding, the pool of potential targets within the non-coding transcriptome is considerably larger than that of protein-coding genes, greatly expanding the landscape of possible dependencies to explore.

For example, as previously described, *PVT1* has been shown to stabilize the MYC protein and enhance its oncogenic activity [76, 174–176]. Because *MYC* paralog amplifications define distinct subsets of SCLC, *PVT1* may represent a context-specific vulnerability in *MYC*-driven SCLC and therefore warrants systematic functional investigation. Similarly, because *NFYC-AS1* is expressed in the 1p34 region, it may be a promising target for *MYCL*-amplified SCLC.

The discovery of novel cancer dependencies and therapeutic targets in SCLC is also often constrained by the available research models (Fig. 5). Most established SCLC cell lines grow in suspension culture and form floating aggregates, which complicates experimental manipulation and downstream assays. To address these limitations, more physiologically relevant models have been developed, including PDX. PDX models preserve the genomic and functional fidelity of patient tumors and better recapitulate the tumor microenvironment and cellular heterogeneity, enabling more accurate therapeutic testing; however, establishing PDX models can be challenging [177] (Fig. 5).

Similarly, organoid systems derived from SCLC tumors provide a three-dimensional culture environment that preserves cellular interactions and NE features. They have been successfully used to study chemotherapy responses [178, 179]. These advanced models offer opportunities to explore novel therapeutic dependencies in a more clinically relevant context.

Moreover, expanding loss-of-function screenings into a multi‑hallmark framework would enable exploration of additional therapeutically relevant phenotypes. For example, drug‑focused CRISPR screens enable the systematic identification of genes whose loss confers drug sensitivity or resistance, therefore providing a powerful tool for mapping gene–drug interactions and predicting therapeutic responses [180, 181]. CRISPR‑based perturbation screens can also be used to dissect the response to immunotherapy or checkpoint‑based therapies by linking gene function to immune‑mediated selective pressure. For example, in vivo CRISPR screens have been applied across diverse tumor models, including melanoma, breast cancer and colon cancer and identified tumor‑intrinsic genes whose loss alters sensitivity to anti‑PD‑1 or anti‑CTLA‑4 treatment within the native tumor microenvironment [182]. Likewise, a genome‑wide CRISPR screen in melanoma cells demonstrated that loss of tyrosylprotein sulfotransferase‑2 (*TPST2*) enhances sensitivity to checkpoint blockade by promoting stronger immune‑mediated tumor control [183]. Integrating these phenotypic screens in SCLC might reveal targets with greater clinical relevance.

Crucially, once therapeutic vulnerabilities associated with specific genomic alterations have been identified, it becomes essential to define biomarkers that can stratify patients for therapy, predict response and longitudinally monitor disease evolution via minimally invasive sampling. In this regard, the analysis of ctDNA and CTCs in the context of liquid biopsy represents one of the most promising approaches for translating genomic biomarkers into the clinic (Fig. 5).

At baseline, liquid biopsy may identify molecularly distinct subsets of SCLC that could benefit from future biomarker-guided therapeutic strategies. For example, should MYC-targeted approaches prove clinically effective, liquid biopsy could enable the non-invasive identification of *MYC*-family amplifications and ecDNA-associated MYC activation for patient stratification and treatment monitoring. Likewise, CNA profiling of CTCs, which can distinguish chemosensitive from chemorefractory disease, illustrates the potential of liquid biopsy-derived genomic signatures as future predictive biomarkers.

During treatment, serial liquid biopsy analyses may enable early assessment of therapeutic response and detection of emerging resistance. Dynamic changes in ctDNA metrics and genome-wide copy-number profiles can reveal molecular responses before radiographic progression. At the same time, genomic and transcriptomic characterization of CTCs may identify resistant cellular populations and capture adaptive processes such as lineage plasticity.

Notably, since synthetic lethality is a gene-network phenomenon, quantifying the expression of other genes within the network associated with the identified genomic targets may provide an alternative approach for identifying clinically relevant biomarkers for patient stratification and treatment response. Realizing this potential will require robust technologies capable of detecting diverse genomic and transcriptomic alterations, including targeted sequencing, shallow whole-genome sequencing, emerging approaches for ecDNA characterization and single-cell profiling of CTCs. However, broader clinical implementation will depend on the standardization of analytical workflows, the harmonization of bioinformatic pipelines, the establishment of clinically meaningful thresholds and prospective validation in large multicenter studies.

## Conclusions

SCLC remains one of the most lethal malignancies, defined by rapid progression, profound genomic instability and early metastatic dissemination. Despite decades of clinical effort, therapeutic advances have been modest, with platinum-based chemotherapy still representing the backbone of treatment and ICIs offering only incremental benefit, largely due to the immunologically cold nature of these tumors. Recent molecular profiling has revealed four major SCLC subtypes and highlighted the dynamic plasticity of their transcriptional states, complicating efforts to exploit subtype-specific vulnerabilities. At the same time, the genetic landscape of SCLC, characterized by pervasive loss of tumor suppressors, widespread CNAs, and ecDNA-driven oncogene amplification, suggests that genomic features may represent more durable therapeutic targets. Liquid biopsy approaches, enabled by the unusually high levels of ctDNA in SCLC, now provide a powerful tool to capture these alterations, including complex structures such as ecDNA, and to use them as biomarkers for patient stratification, treatment response, or monitoring. Complementary strategies, including in silico genetic restraint analyses and in vitro and in vivo genome-wide functional screens, should extend beyond protein-coding genes to encompass lncRNAs, further expanding the search for essential cancer dependencies and opening new therapeutic avenues, such as antisense oligonucleotide–based approaches. Overall, this body of evidence underscores that the identification of actionable vulnerabilities in SCLC will require embracing its biological complexity through the integration of advanced cellular models, genome-scale functional screens in context-specific settings and comprehensive genomic analyses of both tumor tissues and liquid biopsies.

## Data Availability

No datasets were generated or analysed during the current study.

## Abbreviations

cfDNA: Cell-free DNA
CCLE: Cancer Cell Line Encyclopedia
CNAs: Copy Number Alterations
CTCs: Circulating Tumor Cells
ctDNA: Circulating tumor DNA
ecDNA: Extrachromosomal DNA
ES-SCLC: Extensive Stage SCLC
ICIs: Immune Checkpoint Inhibitors
lncRNAs: Long non-coding RNAs
NE: Neuroendocrine
OS: Overall Survival
PDX: Patient-derived Xenograft
PFS: Progression free survival
SCLC: Small Cell Lung Cancer
TF: Tumor Fraction
TMB: Tumor mutational burden
TSG: Tumor suppressor gene
VAF: Variant Allele Fraction
